# Headache during airplane travel (“airplane headache”): first case in Greece

**DOI:** 10.1007/s10194-011-0337-1

**Published:** 2011-04-06

**Authors:** Evangelia Kararizou, Evangelos Anagnostou, George P. Paraskevas, Sofia D. Vassilopoulou, Dimitrios Naoumis, Grigoris Kararizos, Konstantinos Spengos

**Affiliations:** 1 Department of Neurology, Athens National University, Eginition Hospital, 72-74 Vas.Sofias av., 11528 Athens, Greece; 2 Neurological Department, 251 Air Force Hospital, Athens, Greece

**Keywords:** Headache, Airplane flights, Barotrauma, Pathophysiology

## Abstract

Headache related to airplane flights is rare. We describe a 37-year-old female patient with multiple intense, jabbing headache episodes over the last 3 years that occur exclusively during airplane flights. The pain manifests during take-off and landing, and is located always in the left retro-orbital and frontotemporal area. It is occasionally accompanied by dizziness, but no additional symptoms occur. Pain intensity diminishes and disappears after 15–20 min. Apart from occasional dizziness, no other symptoms occur. The patient has a history of tension-type headache and polycystic ovaries. Blood tests and imaging revealed no abnormalities. Here, we present the first case in Greece. We review the current literature on this rare syndrome and discuss on possible pathophysiology and the investigation of possible co-factors such as anxiety and depression.

## Introduction

Every year, over two billion people travel by airplane. Among them, there are thousands who suffer from chronic illnesses with various symptoms, while others develop their symptoms during flight. Over the last years, several cases with airplane flight related headaches appeared. Common characteristics of these patients were the absence of abnormal findings on biochemical and imaging examinations [1–7].

Here, we report on a patient with recurrent headache episodes during airplane flights. We also conducted a brief review on theories about the pathophysiology of this rare condition.

## Case presentation

A 37-year-old healthy woman complained of recurrent headache episodes which occurred during airplane flights. She experienced the first attack 3 years ago during a transatlantic flight. The headache started during take-off and increased gradually to an intense jabbing pain located in the left retro-orbital and frontotemporal area of the skull. The episode lasted 15–20 min and was accompanied by mild ipsilateral tearing. The attack recurred, this time with a milder intensity, during landing. After residing 5 months abroad, she again experienced two attacks on the return flight during take-off and landing. Apart from a slightly shorter duration, she did not notice any differences to the previous episodes. She presented to our headache out-patient clinic because of the regularly occurring headache attacks during her frequent airplane flights over the last months. She had a history of typical tension-type headaches with a frequency of approximately one every 2 months and an average intensity of four on a visual analog scale. She could relate these episodes to stressful periods and the pain is always promptly responding to low doses of common analgesics (acetylsalicylic acid, acetaminophen or paracetamol, mefenamic acid). On the age of 17, after the appearance of hirsutism, acne and menstrual cycle abnormalities, she was diagnosed with polycystic ovary syndrome. She was subsequently put on ethinylestradiol combined with cyproterone acetate which proved effective in suppressing her acne. She stopped taking the drug after 3 years on her own initiative. She was recently seen by an ENT-specialist who did not diagnose a sinusitis or any other inflammation that could cause headaches. An ophthalmological assessment was also normal.

The neurological examination was unremarkable and so were routine blood test. Neuroimaging consisting of brain CT and MRI with contrast revealed no abnormalities. Moreover, her cerebral vessels appeared normal on a subsequent MR-angiogram. The patient was also assessed by a psychiatrist who proposed a low dose antidepressant which the patient declined. Testing for an anxiety and/or depressive disorder revealed a score of 14 on the Hamilton anxiety scale (HAM-A) (mild anxiety ≥14) and 12 on the Hamilton depression scale (HAM-D) (mild depression >7) [8, 9].

She took up to three tablets of paracetamol without any help and was recommended to take a single dose of Ibuprofen, 10 mg/kg/dose, 1 h prior to airplane landing and replay the dose 1 h before descend. Patient referred might help. It is important to mention that, she reports improvement in anxiety after our examination (negative results) and psychological support. She was invited to control visits after her airplane travels.

## Discussion

Atkinson and Lee first reported in 2004 a 28-year-old male patient with intense frontal and retroorbital headache during airplane flights [1]. Later on, several case descriptions with similar symptoms appeared in the literature [2–7]. In 2007, Mainardi et al. [10] formalized a list of diagnostic criteria for this symptom entity:

A. At least 2 episodes fulfilling B, C and D
B. Pain lasting <20 min during airplane flight having at least two of the features given below:
  1. Severe intensity
  2. Pulsating or stabbing quality
  3. Strict unilateral location
  4. Peri- or retro-orbital location, sometimes with involvement of frontal locations
C. No additional symptoms (rarely mild rhinorhea, tearing or face edema)
D. No history or findings of other disorders that could account for the symptoms

The same authors propose an inclusion of this flight related headache in the classification of the International Headache Society due to the steadily increasing number of reported cases in the last years [11].

The present case fulfills all of the above criteria for airplane headache. The physical examination was unremarkable and so was the biochemical and hematological work-up. Brain MRI with gadolinium enhancement was also free of abnormalities. The tension-type headache experienced in the past was clearly distinct from that experienced during flights in terms of intensity, duration and pulsating character. A history of tension-type headache or migraine is also described in many of the airplane headache patients reported in the literature [3].

Dormitz [5] highlights the frequent appearance of airplane headache mainly in young individuals aged between 20 and 40 years, similar to the present case In a recent article, After the first description of airplane headache in pediatric age (Mainardi et al. [6]), Ipekdal et al. [7] described further three cases, Ipekdal et al. described three pediatric cases with airplane headache aged between 12 and 14 years.

Despite the various proposed pathophysiologic mechanisms, the cause and the pathogenesis of airplane flight related headache remains largely unknown. Berilgen and Mungen [3] hypothesize on the rapid change in atmospheric pressure during flight as a possible cause of headache in susceptible individuals, arguing against previously articulated theories such as the influence of high altitude alone. These authors favor a barotrauma-like mechanism, which manifests during the landing procedure of the airplane and is well described in deep water divers. During these situations, the arterial blood pressure of the organism continuously interacts with the rapid alternating environmental pressure outside of the body. Ipekdal et al. noted the importance of an additional ENT assessment in order to rule out ear and nose pathologies that could sensitize trigeminal nerve endings within the facial sinuses. Such a sensitization could result in a deficient adaptation to flight induced changes in atmospheric pressure that could trigger a brief headache attack [7]. The strict retro- or periorbital pain location in the majority of the described cases might also be explained by that hypothesis. Similar headache did not appear in other altitude variation moments, e.g., in mountain trips. Headaches are a common symptom of acute mountain sickness and the International Headache Society has defined a primary headache disorder of high altitude headache as one that develops “within 24 h after sudden ascent to altitudes above 3,000 m,” in association with Cheyne-Stokes respiration, desire to overbreathe, or exertional dyspnea. [12].

Involvement of non-organic, psychiatric factors, such as coexisting affective disorders has not been investigated in this context. Our patient showed both a mild depressive (HAM-D = 12) and a mild anxiety disorder (HAM-A = 14), without having a distinct phobia for airplanes or flying in general. The comorbidity of airplane headaches and (mild or severe) psychiatric disorders should be investigated in larger samples in order to establish a significant association.

We conclude that knowledge of the existence, the characteristics and the age group of flight related headaches is critical for diagnosis. As Evans et al. [2] noted airplane flight headache “might not be all that rare but just rarely reported”. Detailed clinical and laboratory work-up, as well as gathering of information about possible co-factors such as anxiety and depression, might help elucidate the pathophysiologic mechanisms and introduce successful therapeutic strategies in the future.
